# Media and government framing of asylum seekers and migrant workers in Canada during the COVID-19 pandemic

**DOI:** 10.1007/s11159-021-09932-8

**Published:** 2022-01-26

**Authors:** Michelle Stack, Amea Wilbur

**Affiliations:** 1 Department of Educational Studies, Peter Wall Institute for Advanced Studies, University of British Columbia, Vancouver, BC Canada; 2 Faculty of Professional Studies, University of the Fraser Valley, Abbotsford, BC Canada

**Keywords:** Media, Refugees, Asylum seekers, Canada, Social movement education, Critical discourse analysis, Adult education

## Abstract

One understudied area of adult education and lifelong learning is the role of media as educator and policy player. This article describes how the authors used *critical discourse analysis* to examine how asylum seekers, migrant workers and their advocates have challenged long-standing discursive framings of them as benefactors of Canadian generosity, criminals, burdens or victims – during the first ten months of the COVID-19 pandemic. The analysis points to the difficulties of navigating media engagement to advocate for individuals facing deportation from Canada, while also attempting to challenge the dichotomy of people seen either as worthy of dignity (those who work for low pay and in dangerous conditions to care for Canadians) or as unworthy (those who work on farms or who are not able to work). However, it also reveals the potential for critical lifelong media education to inform the work of adult educators across classroom, labour and social movement contexts to disrupt exclusionary and oppressive media and government narratives.

## Résumé

Le cadrage médiatique et gouvernemental des demandeurs d’asile et des travailleurs migrants au Canada pendant la pandémie de COVID-19 – Le rôle d’éducateur et d’acteur politique des médias est un domaine peu étudié de l’éducation des adultes et de l’apprentissage tout au long de la vie. Cet article décrit comment ses autrices ont utilisé *l’analyse critique du discours* pour examiner la façon dont les demandeurs d’asile, les travailleurs migrants et leurs défenseurs ont remis en question, au cours des dix premiers mois de la pandémie de COVID-19, les cadres discursifs traditionnels qui faisaient d’eux des bénéficiaires de la générosité canadienne, des criminels, des fardeaux ou des victimes. Cette analyse met en lumière les difficultés à diriger l’engagement médiatique pour défendre les personnes confrontées à la déportation du Canada, et elle essaye en même temps de remettre en cause la dichotomie de la perception des individus considérés soit comme méritant le respect (ceux qui travaillent en étant mal rémunérés et dans des conditions dangereuses au service des Canadiens), soit comme ne le méritant pas (ceux qui travaillent dans des fermes ou qui ne sont pas capables de travailler). Néanmoins, elle révèle aussi les possibilités qu’offre l’éducation médiatique critique tout au long de la vie pour orienter le travail des éducateurs d’adultes dans les contextes des cours en classe, du travail et de mouvements sociaux afin de rompre avec les discours exclusifs et oppressifs des médias et du gouvernement.

## The story of a welcoming country

### Michelle's story

*I remember back in the early 1990s teaching a course on needs assessment for EAL [English as an Additional Language] teachers in Canada. At the time, there was a lot of negative media attention about Islam and Somali refugees. When I asked teachers what they knew about the context of their students, they repeated negative media coverage about Somalis. It became clear how they had constructed ideas about their students based not on experience with people from a region or by reading authors from the region but from media accounts. How, I wondered, does this impact the way teachers construct ideas about students and their needs before even knowing their names?*

### Amea's story

*Before working at the University of the Fraser Valley in Canada, for several years I was a manager at a non-profit organisation that worked with migrant women. In 2016, with a large number of Syrians arriving in Canada, the organisation was contacted regularly by media wanting a “sound bite” about the experiences of refugees. I was keenly aware that the stories I was reading and listening to in the legacy [traditional] media did not represent the complex experiences of the women I worked with. It left me wondering how media engage with migrants, whose stories are being told, why certain stories are told and who is telling them. These questions led me to contact a media outlet that wanted a more collaborative and reciprocal relationship. I met with two journalists who wanted to tell stories that did not perpetuate racist and xenophobic ideas about migrants. Together*, The Discourse *[an alternative online media publication], journalists, 30 new asylum seekers and migrants to Cana-da, settlement workers and academics developed a toolkit offering tips and guidance for refugees interested in sharing their perspectives, and for journalists who want to do a better job of talking about refugees.*

During the COVID-19 pandemic, there has been media coverage of asylum seekers in Canada who are being threatened with deportation despite putting their lives at risk to care for Canadians in hospitals and care facilities such as nursing homes for elderly citizens. As the stories that introduce this article show, we both have a long-standing interest in examining the framing of asylum seekers and migrant workers, as well as what can be done to better inform the narratives. In this article, we analyse media and federal government framing of migrant workers and asylum seekers^1^ in Canada, beginning with the Canadian government’s announcement of emergency measures to respond to COVID-19 in March 2020. The work of asylum seekers, migrant workers, and their advocates in producing and disseminating counter-narratives is evident. We also focus on answering the following research questions:

1. *What discursive frames are evident in the construction of ideas about asylum seekers and migrant workers who provided care and services to Canadians during the first ten months of the COVID-19 pandemic?*
2. *How are the skills and abilities of migrant workers and asylum seekers represented?*

Note that in this article we refer to media as plural. For the purposes of our research, it encompasses legacy media^2^ and social media.

## Representations of migrants and asylum seekers

As Maureen Kihika states,

Despite seeming inclusive, Canada’s history as a white settler society functions on the basis of a contradicted identity: on one hand, multicultural policy generates a national pride of “welcoming‐ness”, while, on the other hand, capitalist pursuit – fundamental to its identity formation – reinforces racialized poverty as a structural mechanism for reproducing white privilege and exceptionalism (Kihika 2020, p. 713).

Boaventura de Sousa Santos argues that the project of “Western modernity” was based on the formation of an “abyssal line”. That which is deemed true and factual is “this side of the line”, while what is on “the other side of the line” is made invisible (Santos 2007, p. 45). Government policies are constructed in ways that assume that migrants come with deficits. As Hongxia Shan (2012) notes, migrant workers who come to Canada are expected to attend workshops and assimilate into what is assumed to be the “right” and “Canadian” way. Shibao Guo observes that

a racialized regime of skill has become a social engineering project for manufacturing normative, white, and docile corporate subjects who are expected to think, act, and behave like a “real” Canadian. In this view, a racialized regime of skill has functioned as an assimilation tool for moulding racialized minorities into Canadian norms and workplace cultures (Guo 2015, p. 247).

Authors of several studies have noted how refugees are often framed as “criminals”, “a wave”, “a crisis”, “the other/outsider” or “victims” (Lekić-Subašić 2018; Quinsaat 2014; Nerghes and Lee 2019, p. 276). Another typical frame is the victim who is “saved” by nations that are seen to be taking too many refugees (Joris and De Cock 2019). Thouraya Zheni’s (2019) analysis of Syrian refugees demonstrates the pervasiveness of media frames of “crisis”, “asylum”, “conflict”, “displaced” and “risk” in describing asylum seekers. Andrea Lawlor’s (2015) cross-national study analysed media coverage in Britain and Canada from 1999 to 2013. It demonstrated how Canadian coverage is often motivated by specific incidents such as a terror attack. These studies illustrate how legacy media and social media create, amplify and transform narratives by creating feelings of fear (based on threats to security and safety), apprehension or even empathy towards asylum seekers (Esses et al., 2013).

As Guo points out, migration has become key to a flexible workforce, “to be deployed at the discretion of the host country” (Guo 2014, p. 485). Unfortunately, there is a devaluing of the prior learning and work experience that migrants bring to Canada. Instead, they are seen as having deficits and therefore needing remedial training, both to accept downgraded jobs and on how to fit into Canadian workplaces. Irrespective of their existing skills and expertise, migrants’ ethnic origin, their skin colour (as well as gender and class etc.) lead to their racialisation, devaluing both their knowledge and also their multilingualism (Maitra and Guo 2019). Drawing on the work of Ramón Grosfoguel and Ana Margarita Cervantes-Rodriguez (2002), Srabani Maitra and Shibao Guo argue that there is a need for “a second decolonization in the context of lifelong learning, a more profound one than the ‘juridical-political decolonization’” (Maitra and Guo 2019, p. 12) to address global inequity and power asymmetries grounded in the current capitalist world system. We argue that critical media education is key to this decolonising work.

## Critical media education and social movements

Calls for lifelong media education are long-standing; it was recommended, for example, in 1960 by the Second International Conference on Adult Education (CONFINTEA II),^3^ organised by the United Nations Educational, Scientific and Cultural Organization (UNESCO). Two decades later, the *Grünwald Declaration* on media education (UNESCO 1982) also called for comprehensive media education at all levels, including in adult education. Critical media education is based in a practice of deconstructing and working to dismantle oppression. Pierre Walter notes that “social movements create counterpublics and counterknowledges challenging the hegemony of dominant corporate and state discourse” (Walter 2007, p. 259).

The past five years have led to renewed interest in the role of media in promoting dominant corporate and state agendas. The election of xenophobic figures (such as Trump in the United States [US], Modi in India and Duterte in the Philippines), for example, points to the role of social media and legacy media in fuelling fires of hate. However, Elizabeth Roumell and ArCasia James-Gallaway (2021) also describe how the Black Lives Matter movement made tactical and sophisticated use of social media to tell the stories of Black people, and to organise resistance to continued corporate and state oppression.

Elizabeth Tisdell speaks specifically about her engagement with critical media education as part of an important but under-theorised practice in adult education. She draws on the work of Paulo Freire and Donaldo Macedo to “read the world as well as the word” (Freire and Macedo 1987). Her work points to how media education is part of understanding how popular culture can “both resist and reinforce the interest of dominant culture” (Tisdell 2007, p. 6).

## Methodology

As mentioned at the beginning of this article, the purpose of our study was to investigate media and federal government framing of migrant workers and asylum seekers. We examined major media outlets’ coverage of asylum seekers and migrant workers in Canada – as well as news statements and press releases from the Canadian Ministry of Immigration, Refugees and Citizenship (IRCC) – from 1 March to 10 December 2020. We chose this period because it included the beginning of the COVID-19 pandemic, when media and policy representations of asylum seekers and migrants were extensive.

We used *critical discourse analysis* (CDA), a social practice approach to language and communication which assumes that discourse practices mediate between texts and society or culture (Fairclough 1993). Marianne Jørgensen and Louise Phillips argue that it is

through analysis of intertextuality [that] one can investigate both the reproduction of discourses whereby no new elements are introduced and discursive change through new combinations of discourse (Jørgensen and Phillips 2002, p. 7).

As we examined media coverage about asylum seekers, we sought to understand the *interdiscursivity* that “occurs when different discourses and genres are articulated together in a communicative event” (ibid., p. 73). This body of media coverage alongside news statements and IRCC press releases provided sample material for us to consider how government and media texts are written with particular audiences in mind. CDA affords an opportunity to develop a partial understanding of how social structures are created and recreated.

Discourses bring categories such as “refugee” into being. Who is categorised as a refugee versus someone displaced by climate change is based on who has the power to speak and define who is a refugee. As Sabine Lehner and Markus Rheindorf remind us,

the degree to which this body is demarcated, that is, bordered, is an integral component of its discursive construction rather than a natural trait – the often-claimed “naturalness of borders” is itself discursively produced (Lehner and Rheindorf 2018, p. 42).

Those seen as experts are positioned to be heard and to discursively construct categories of people (Potter and Wetherell 1987).

In our analysis, we examined who speaks in articles about asylum seekers and migrant workers as evidence of discursive struggles between governments and advocates as they engage with media to garner legitimacy for their framings. We limited our study to the analysis of printed texts available through the Canadian Broadcasting Corporation (CBC) website and the Canadian Newsstream database. For the latter, we focused on the following legacy media and newspapers: the *Globe and Mail,* the *National Post*, the *Toronto Star* and the *Vancouver Sun.* We decided to start with legacy media and newspapers because these outlets still carry immense weight in setting the parameters for debate in social media and in influencing government policy (Nerghes and Lee 2019). Through the CBC website and Canadian Newsstream database, we located and analysed a total of 49 articles (published between 1 March and 30 December 2020) that focused on asylum seekers and migrant workers. We also analysed six statements on the IRCC website during the same period.

## Findings

The first part of our findings focuses on how the Canadian government and politicians represented asylum seekers and migrant workers, and how media covered these statements. The second part of our findings focuses on how asylum seekers and migrant workers challenged these representations through their lived experience.

### Discursive frames: guardian angels and criminals

Several of the articles we located refer to asylum seekers who work in long-term care facilities in Canada for little pay as “guardian angels”. In the first paragraph of one article published on 8 June 2020, *Globe and Mail* health reporter André Picard tells his readers that

They call them the “guardian angels”, the thousands of personal-support workers (PSWs), orderlies, cooks and janitors who have been toiling for months in Quebec’s beleaguered and often overwhelmed long-term care homes during the COVID-19 pandemic. Almost all of them are women, many from racialized communities … despite doing essential work that no one else would and literally putting their lives at risk, juggling multiple part-time gigs for as little as $13 an hour, many of these front-line workers could face deportation. That’s disgraceful, and un-Canadian (Picard 2020).

This article points to the contradiction in seeing the workers as guardian angels – yet treating them as outsiders. Picard also notes that most of these workers are women and racialised. Key to his message is that the common trope of Canada being welcoming makes this exclusion “un-Canadian”. Picard expands the frame beyond asylum seekers as objects of charity or as criminals; however, he maintains the deserving (those who risk their lives for Canadians) and undeserving asylum seekers frame. His article points to the tension between the Quebec government and asylum seekers:

In 2017 and 2018, more than 37,000 people made an “irregular” border crossing and requested asylum in Canada. Most of them simply trudged up Roxham Road in Saint-Bernard-de-Lacolle, Que., exploiting a loophole in the Canada-U.S. Safe Third Country Agreement, which said they could only be turned back at official border crossings (ibid.).

The language of “exploiting a loophole” or “simply trudg[ing] up” a road reinforces an image of undeserving asylum seekers. A *Toronto Star* article (Ballingall 2020) published on 14 August 2020 quotes the then Minister of Immigration, Refugees and Citizenship, Marco Mendicino, extensively. In the article, Mendicino called healthcare workers “heroes” and promised that “such an extraordinary service to Canadians should be answered with an extraordinary, ‘one-time’ pathway to permanent residency”. He explained that this group is viewed differently to other asylum seekers: “What makes this group so unique and so special is the adversity that they had overcome just to get here.” It is unclear how other asylum seekers were able to get to Canada without adversity. How did he determine that only those he deemed heroes faced adversity? The article continues quoting him:

“And despite the fact that they themselves were very vulnerable, [they] put themselves at a high risk to help others in their community,” he said. “Even though they don’t possess Canadian papers that give them permanent resident status or immigration status, they demonstrated a uniquely Canadian quality by looking out for one other” (Ballingal 2020).

The Minister’s statement implies that caring for others is something uniquely Canadian and that, somehow, people who are not yet Canadian have proven they too can care so the government will make a special case for them. Nowhere in the article is the Minister asked how he determined that Canadians are unique in caring for others or the ethics of requiring people to put their lives at risk to be considered for permanent residence.

Statements and news releases from the Government of Canada provide a picture of a welcoming Canada. A statement by Minister Mendicino (IRCC 2020a) issued on 10 December 2020 asks Canadians to remember “Operation Syrian refugees”.^4^ Using the term “operation” paints a picture of military precision deployed to carry out “a massive humanitarian effort [which] showed the strength of our country and the depths of our compassion” (ibid.). The reader is also informed that “In 2018 and 2019, Canada was the top resettlement country globally” (ibid.). Indeed, Canada has been praised by the United Nations (UN) for resettling refugees; however, of the five countries that host the highest number of refugees (Turkey, Colombia, Pakistan, Uganda and Germany) three are economically marginalised, developing countries (UNHCR 2021). Most refugees move to other poor nations and, according to Amnesty International, only half a per cent of refugees were resettled in 2019 (AI 2019).

Messages of gratitude to asylum seekers who have been heroes and angels to Canadians during COVID-19 are present on the Government of Canada website, but so too are dire warnings to prospective asylum seekers. In a news item published on 5 February 2020, under the prominent heading “Irregular border crossings – What is Canada doing?”, readers are told:

Canada remains a fair and welcoming country but will not tolerate abuse of that generosity. The Government of Canada is taking additional steps to ensure the integrity of our immigration system. Irregular border crossers will have their claims heard quickly, and, if they are found not to be in need of Canada’s protection, will be removed quickly (IRCC 2020b).

In this statement, people are transformed into “irregular border crossers”. There is an assumption that irregular border crossers or abusive rule-breakers have created the need for a strategic plan to deal with “an influx of irregular arrivals via the Canada–U.S. land border” (ibid.).

In an IRCC webpage published on 23 July 2020 titled “For asylum seekers: What you need to know”, we found this statement:

Canada respects its international obligations toward those who genuinely need help and protection. However, we must also make sure that all laws are followed to protect the safety, security and health of Canadians. … Our rules-based system will determine the validity of your claim … You’ll be removed from Canada if you don’t have a legitimate claim (IRCC 2020c).

The Federal Court’s decision on the “Safe Third Country Agreement” between Canada and the US was released in July 2020 (IRCC 2020d). It was the result of an appeal by the Canadian Council for Refugees and Amnesty International that was launched in 2017. The reader is not told that the Federal Court decision was based on evidence that people fleeing domestic and gang violence were particularly at risk of deportation from or detention in the US since the election of Donald Trump as President. Several cases were presented to the Federal Court of women fleeing violence, as well as evidence of forced sterilisation in US prisons.

The choices governments make in creating or mitigating vulnerability through policy are not questioned. Most of the articles we examined report low pay and dangerous jobs as the natural order of things for asylum seekers and migrant workers. There has been little querying about the absence of policies to ensure fair remuneration and safe work conditions. Guardian angels and heroes are represented as good people, but rarely are the skills and education these workers bring to their workplaces mentioned.

### Disease carriers and “off-the-books” workers

Headlines often reinforce the disease-carrying trope (Levitz and Kestler-D’Amours 2020). However, the content of articles is sometimes more nuanced; for example, a CBC headline published on 2 July 2020, “How undocumented migrant workers are slipping through Ontario’s COVID-19 net: 2,000 off-the-books foreign labourers complicate pandemic battle in Windsor-Essex” (Gatehouse 2020) appears to blame “off-the-books” (unofficial) workers as the problem. In contrast, the article includes stories of workers balancing their health and making money to support large families in their home countries. Again, the question is not asked as to why so many people are in the predicament of having to take these risks. CBC Windsor quotes a local Conservative Member of Parliament (MP) who, throughout the piece, blames “these workers” for the rise in COVID”.

Dave Epp, Conservative MP for Chatham-Kent – Leamington believes these workers are playing a key role in the spread of the coronavirus in Leamington and Kingsville. Those regions have been held back by the province from moving into Stage 2 of reopening due to the high rates of the disease (CBC 2020a).

In the article, published on 6 July 2020, “illegitimate workers” are blamed not just for the conditions of the farms they work on but the inability of the entire province to deal with COVID-19. The piece does not quote workers or advocates. Epp’s position is stated as fact, yet no evidence is provided.

On 20 July 2020, *The Guardian* published an article concerning the deaths of three migrant workers in Ontario since the COVID-19 pandemic began. It also critiques Canada’s treatment of workers. The article describes a farm that allowed Canadian workers freedom of movement but told migrant workers they were not allowed off the property. Prime Minister Justin Trudeau is cited:

“It’s obvious that we need to do a better job of ensuring that the rules are followed for our temporary foreign workers in Canada,” Trudeau said in June after the deaths of three workers who contracted Covid-19. “Every single person who works in Canada deserves to do so in a safe environment and unfortunately that hasn’t always happened” (Beaumount 2020).

In this article, the workers are labelled as “ours”, suggesting that “we” have the right to tell them how to behave and to blame them for illness and death should they not follow the rules. The crowded living conditions, low pay and inequity of allowing Canadians to move about – but not migrants – are absent in the discussion. Again, there is an implicit assumption that non-Canadians are disease carriers and need to be prevented from spreading illness.

On 18 December 2020, International Migrants Day, a federal government press release stated:

We are all too aware of the challenges and difficulties migrants face on a daily basis. On this day, we take a moment to celebrate their courage and resiliency (IRCC 2020e).

Readers are assured that Canada does not just take “anybody”: “We continue to welcome newcomers, who bring the skills and commitment that our economy and our communities need to thrive” (ibid.). Throughout the government statements we reviewed, the focus is on Canada’s economic needs. “Good” newcomers are represented as good for the economy and bringing a commitment to “our” communities. Until newcomer workers are accepted, they are “ours” but not part of “our” communities.

Absent is the role of Canada as a factor in who becomes an asylum seeker. The UN and other groups, for example, have repeatedly expressed concern about Canadian companies implicated in human rights abuses and asked Canada to develop policies “to protect against business-related human rights abuses and promote effective business respect for human rights …” (UN working group on business and human rights, quoted in HRW 2019, p. 123). On the one hand, for example, the Canadian government expressed outrage at Saudi Arabia for the trial of human rights activist Loujain al-Hathloul and the violation of women’s rights. Global Affairs Canada spokeswoman, Angela Savard, said in an e-mail statement regarding her case: “True to our democratic values and principles, Canada will always stand with human rights activists and defenders, around the world” (Haig 2020). On the other hand, however, in 2019 the Canadian government sold nearly three-billion dollars’ worth of military equipment to Saudi Arabia (Cecco 2020).

### Asylum seekers and advocates educate through media

The treatment of asylum seekers caring for Canadians during the COVID-19 pandemic garnered national and international media attention after the Quebec newspaper *La Presse* reported the death of asylum seeker and personal care attendant Marcelin François on 14 April 2020. Yves Boisvert (2020), writing for *La Presse*, notes that François and his family were denied refugee status because “When you take refuge only from poverty, you are not a refugee within the meaning of the law”. Similar to many of the articles we analysed, this one uses the language of war: “He was the unknown soldier to health combat, you know, all those anonymous people in the mass graves of history, ‘dead for the homeland’? A homeland he almost touched …” (ibid.).^5^ This statement is complex. On the one hand, the author notes how the power to define who is and who is not a refugee can have a deadly consequence. On the other hand, however, the language of “homeland” erases the reality that Canada is occupying the homelands of people who are indigenous to Turtle Island.

A *Washington Post* article published on 12 June 2020 quotes an asylum seeker who was a doctor in Haiti and is now a nursing assistant in Quebec. She spoke about not considering leaving her job despite the risk, yet journalist Amanda Coletta notes,

Ledan is one of at least several hundred asylum seekers risking their lives, and in some cases dying, for as little as $10 an hour in the trenches of Canada’s coronavirus fight, working the “essential” jobs few Canadians want, even as their own futures in the country remain uncertain (Coletta 2020).

The author explains that Prime Minister Trudeau is under pressure to respond and has plans to do so, but advocacy groups are critical of the lack of progress and respect accorded to asylum seekers who have done work that Canadians do not want to do. Conservative MP Peter Kent is quoted as saying:

“It’s admirable that they took these jobs before the COVID crisis, and it’s admirable that they have, in most cases, stayed in these positions to care for the most vulnerable, … [b]ut we don’t believe that that should be a shortcut to Canadian permanent residency … It would be, I believe, an unacceptable precedent” (ibid.).

The politician does not appear to be challenged to explain why it is an “unacceptable precedent”, nor is he asked to respond to the critiques of advocates. The article ends with the words of Ledan and her call for change.

She has a message for Trudeau and Legault as they debate regularizing her status. “It’s not a loss, but a win for them”, Ledan said. “We are loyal people and we are ready to continue to work for a better Quebec and for a better Canada” (ibid.).

An article by Martin Lukacs, published by the CBC on 16 June, stands out for highlighting the contradictions in Canadian beliefs about being a welcoming country and the reality for asylum seekers in terms of exclusion and criminalisation. He notes that deportation targets have gone up by 30 per cent, and that there is a national target of 10,000 deportations a year under the Trudeau government which “proclaims its friendliness to refugees and migrants” (Lukacs 2020).

It’s hard to miss the irony: while some asylum seekers do jobs lauded as “guardian angels” others are being subjected to treatment lifted straight from the criminal justice system … thousands of undocumented immigrants, including children ... in jail-like detention centres on the outskirts of Montreal, Vancouver and Toronto, or in maximum security prisons. Canada remains one of the only countries in the world that does not put limits on the time that non-citizens can spend in detention without trial (ibid.).

Lukacs clarifies that most are in prison not because they are charged with a crime but because of “irregularities in their refugee or immigration claims”. He notes the hunger strikes that occurred as COVID-19 spread, and the pressure that may have led to the release of some asylum seekers from jail. He also describes how many were forced to wear electronic monitoring devices as a condition of their release. In some cases, media reports provide examples of people being detained and deported or having to wear electronic monitoring bracelets that sometimes malfunctioned in public (Lukacs 2020). Lukacs paints a vivid picture of people who have committed no crime but are treated as dangerous offenders. Similarly, a CBC article published on 20 July 2020 (Cabrera 2020) quotes Frantz André, a founder of the Action Committee for People Without Status, who explained that he receives hundreds of calls a day from asylum seekers. “A lot of them are depressed to the point of being suicidal” (ibid.) due to the length of time (on average 30 months) it has taken the government to make a decision about their immigration status.

Several media pieces covered protests organised by migrant workers, asylum seekers and their advocates, drawing attention to their pleas. The CBC reported on a rally held a week before World Refugee Day (which is on 20 June). The protesters made sophisticated use of visuals, personal stories and statistics. They stated both the problem (asylum seekers and workers are treated as disposable) and a simple solution (treat them as humans and Canada will benefit). For example, a young man of colour, wearing a mask to protect himself and those around him against COVID-19, was holding up a sign stating “Canada is the most welcoming country for refugees”, which is juxtaposed with the headline “Dozens gather downtown to demand permanent status for refugees amid COVID-19”. The article (Ricci 2020) quotes Sally Atta, a shelter worker and refugee claimant, who expressed her pleas in these words:

“We are asking the Canadian Government, please, have mercy. It doesn’t matter the sector we are working in … I work six days a week. And I am sure this is the same for a lot of refugee claimants out there. We have put our life on the line during this COVID pandemic and we are praying” (ibid.).

The article ends by quoting Prime Minister Trudeau, who stated that the government cares and is doing all it can. Absent is a response from Trudeau about the experiences of asylum seekers, as cited earlier in the article (ibid.).

A Canadian Press story published on 15 August 2020, which was picked up by print and television outlets, quotes Doll Jean Nguessan Bi, another asylum seeker, who stated that “Friends who worked with me in security that abandoned [their posts] because they were afraid of getting infected. But I stayed” (Kestler-D’Amours 2020). Repeatedly, asylum seekers seem required to demonstrate that they have put their lives on the line to be deemed eligible to be a legal resident of Canada.

On 23 May 2020, a CBC piece drawing on files from *La Presse* stated that Marcelin François’s “death was first reported in La Presse and fuelled calls for the provincial and federal governments to guarantee asylum seekers like him would be able to stay” (Stevenson 2020). There is little acknowledgement of the extensive labour of advocates and asylum seekers to educate journalists and change policies. In reporting on a rally that involved over 1,000 people, one article states:

It’s the second time in two weeks that the group Debout pour la dignité has organized a march from Trudeau’s riding office [electoral district], located in Montreal’s Villeray neighbourhood. After their first demonstration, Quebec Premier François Legault said he would ask Immigration Minister Simon Jolin-Barrette to look at the asylum claims of those working in CHSLDs [residential and long-term care centres] on a case-by-case basis (CBC 2020b).

The article shows an image of a woman holding up a poster in Creole that is translated to mean “We will not die for your thank you. We will not die in vain”. Wilner Cayo, the president of Debout pour la dignité, also drew attention to the “deeply racialized” nature of discrimination against asylum seekers, noting that “most of the asylum seekers working in essential jobs in Quebec are black, many of them from Haiti”. Cayo further commented that “the conditions under which many of the asylum seeker orderlies work amount to modern slavery” (ibid.). Here, Cayo provides powerful evidence to counter the mythology taught to children and adults through textbooks and media about a fair and equitable Canada. The article concludes by stating that some opposition members of the National Assembly of Quebec (the province’s legislative body) did attend the rally. However, there appears to be no attempt to pressure the Canadian or Quebec governments to respond to asylum seekers and their advocates.

A Canadian Press article picked up by the CBC on 19 September 2020 is titled “BC migrant, undocumented workers rally for the Permanent Residency Program”, with the subheading “The pandemic has also exposed the extent to which these essential workers do not enjoy essential rights” (Smart 2020). The article quotes several asylum seekers and advocates and mentions government plans to improve the treatment of migrant workers. It includes a Zoom screenshot of workers in their respective locations with their fists in the air at a virtual Amnesty for Undocumented Workers Campaign led by the Migrant Workers Centre in Vancouver.

In an opinion piece published on 16 September 2020 in the *Ottawa Citizen*, Dr Meb Rashid, director of the Crossroads Clinic at Women’s College Hospital, and Maureen Silcoff, president of the Canadian Association of Refugee Lawyers, point to the contradiction of Canadian policies around COVID-19. They note that Canada had been opening up to hockey players and some international students but that it continued to restrict refugees’ admission (Rashid and Silcoff 2020, B2).

Asylum seekers were sometimes referred to as “guardian angels” by politicians; however, at the same time they were continually denied healthcare when they contracted COVID-19 on the job. An article written by the Canadian Press was picked up by several outlets in Canada, including the *Globe & Mail, Toronto Star*, and several local, international and online outlets. The article, published on 18 May 2020, quotes an advocacy organisation and Carole Ze Benedicte, an asylum seeker from Cameroon. She echoed the inconsistencies: “When we die at the front lines, we’re called guardian angels”, she said. "But when we need to be treated on equal footing, we’re not guardian angels. We’re nobody, we’re invisible” (Lowrie 2020).

Another article, published on 26 May 2020 in the *Toronto Star*, is titled “Her job is to care for the vulnerable during the pandemic”*.* It speaks to the contradictions in Canadian government statements of welcome and policies of deportation. Among those to be deported, once COVID-19 is under control, is Amaka Oregbemhe who “does what Prime Minister Justin Trudeau calls ‘heroic work’” (Ballingall 2020). She is a mother of three working

on the front lines of the COVID-19 pandemic, picking up night shifts as a personal support worker at a group home for people with disabilities on top of her full-time hours in supportive housing for seniors … But now, two years after arriving in Canada seeking asylum from Nigeria, the family faces the prospect of being sent back after their refugee claim was denied (ibid.).

The trope of asylum seekers who accept being treated with a lack of dignity and respect has been repeatedly challenged by activists and asylum seekers themselves. This was particularly evident after the first reported death from COVID-19 of asylum seeker Marcelin François. Activism and media coverage appeared to create pressure on the government to act, and there were some policy changes as a result. For example, in the summer of 2020, Federal Immigration Minister Marco Mendicino announced two new temporary immigration programmes for asylum seekers who worked as orderlies, nurses and service aides.

What is clear from our analysis is that the protests that were covered by media were organised and strategic. Prominent visuals, such as images of asylum seekers who have died, were used. Asylum seekers and advocates had clear messages. The protests appeared to garner attention and support from politicians and journalists. For example, an article published on 4 May 2020 in the *Toronto Star* stated that

Dozens of Canadian elected officials have signed a petition urging the federal government to extend COVID-19 financial support to all undocumented migrants in the country (Keung 2020).

Then on 23 November 2020, the Federal Government, through a statement titled “Temporary public policy to facilitate the granting of permanent residence for certain refugee claimants working in the health care sector during the COVID-19 pandemic”, appeared to respond to protests by announcing a pathway to permanent residency for some asylum seekers. Of note is that the statement acknowledges the influence of media and advocacy in the policy.

Recent media reports and stakeholder interventions have drawn attention to the extraordinary contribution of refugee claimants working in Canada’s healthcare sector during the COVID-19 pandemic, particularly in long-term care centres in Quebec (IRCC 2020).

In the statement, Minister Mendicino said, “the program is a way to thank those who ‘put themselves at the greatest risk’ of contracting the coronavirus”. However, only those meeting various criteria for permanent residency, “notably health and safety requirements, who applied for asylum before 13 March 2020, and [who] worked in patient care at a health institution for at least 6 months qualify” (ibid.).

## Conclusion and implications

Absent in the media coverage we reviewed was any questioning of Canada’s right, as occupiers of Indigenous lands, to make moral claims around who is considered to be an irregular or illegal asylum seeker. How Canada is implicated in the need for people to flee is also not mentioned, nor is the connections between women’s rights, Canadian military equipment and displacement. The narrative of the Canadian government appears to be simple: Canadians are generous and humanitarian, and they do not tolerate racism. However, in the media we analysed there were moments where another story was told. Tropes of heroes and guardian angels were used in much of the coverage, but absent was attention to the skill and education that asylum seekers and migrant workers bring to Canada.

Asylum seekers, migrants and their advocates were engaged with as informed sources for articles. However, we note that the dominant frame of the helpful asylum seeker is one who benefits the economic needs of Canadians. We recognise that for those trying to ensure some justice for some asylum seekers, the frame of the guardian angel or hero is strategic, but it also leaves intact the notion of some people being more worthy and useful than others within Canada’s capitalist system. The struggle to find the narrow cracks to expand public policy beyond legal and illegal people is challenging to navigate.

Because media reporting tends to focus on events and episodes, coverage of systemic and institutional racism and other forms of discrimination is harder to achieve. However, as some of the coverage we analysed demonstrated, it is not impossible. Advocacy organisations, including the Canadian Council for Refugees and the Migrants’ Rights Network, leveraged the moment of crisis brought on by the COVID-19 pandemic to push for a frame that acknowledges the life-saving work that some asylum seekers do. It still leaves out the years of work asylum seekers have done before the pandemic – and that even though there is a new pathway for a select few, most are still excluded (e.g. essential food service workers and security guards).

It is through media that racism is too often amplified; however, as we see in the case of COVID-19, asylum seekers and their advocates do push back. This is not to say that injustice can be fought merely through media engagement, but that media education is vital for providing tools for people to expand the parameters of debate and representation by those who are most impacted by racist and xenophobic representations of their experience, needs and aspirations. We propose pedagogical spaces for critical transnational lifelong media education that engages with the work and specific campaigns of social justice movements. Critical lifelong media education provides a pedagogic approach to understanding how adult educators, whether in formal classes or social movements – and government policymakers – can come to recognise or misrecognise migrants and asylum seekers, and how solidarity can help disrupt exclusionary and oppressive media and government narratives.

## Footnotes

1 In this article we use the terms *asylum seekers* and *migrants*. Asylum seekers include those who have officially been granted refugee status and those seeking such status.

2 Legacy media refers to traditional mass media, such as print newspapers, television stations and radio broadcasting.

3 The acronym derives from the conference’s French designation: CONFérence INTernationale sur l’Education des Adultes. “The conference, held at Montreal from 22 to 31 August 1960, had as its theme 'Adult education in a changing world'. It offered educators an opportunity of reviewing the development of education over the previous decade and of formulating plans to meet future needs. It provided, in addition, a firm basis for international co-operation in the advancement of national and international adult education activities” (UNESCO 1963, Preface).

4 Mendicino’s statement was issued on the fifth anniversary of this campaign. “Operation Syrian refugees” refers to an initiative which lasted 100 days, from November 2015 to February 2016, during which private sponsors, non-governmental organisations and Canadian provincial, territorial and municipal governments worked together to welcome more than 25,000 Syrian refugees.

5 We translated these two statements into English for the purposes of this article. The original French versions are “*Quand on se réfugie seulement de la misère, on n’est pas un réfugié au sens de la loi*”; and “*Il est au combat sanitaire ce qu’était le soldat inconnu, vous savez, tous ces anonymes dans les fosses communes de l’histoire, ‘morts pour la patrie’ ? Une patrie qu’il touchait presque …*”
